# What Does Imaging Reveal About the Pathology of Amyotrophic Lateral Sclerosis?

**DOI:** 10.1007/s11910-015-0569-6

**Published:** 2015-05-26

**Authors:** Martin R. Turner, Esther Verstraete

**Affiliations:** Nuffield Department of Clinical Neurosciences, University of Oxford, Oxford, UK; University of Utrecht, Utrecht, Netherlands; John Radcliffe Hospital, West Wing Level 3, Oxford, OX3 9DU UK; University Medical Center, Heidelberglaan 100, Utrecht, Netherlands

**Keywords:** Motor neurone disease, Magnetic resonance imaging, Positron emission tomography, Diffusion tensor imaging, Functional imaging, Cortical thickness

## Abstract

Amyotrophic lateral sclerosis (ALS) is now recognised to be a heterogeneous neurodegenerative syndrome of the motor system and its frontotemporal cortical connections. The development and application of structural and functional imaging over the last three decades, in particular magnetic resonance imaging (MRI), has allowed traditional post mortem histopathological and emerging molecular findings in ALS to be placed in a clinical context. Cerebral grey and white matter structural MRI changes are increasingly being understood in terms of brain connectivity, providing insights into the advancing degenerative process and producing candidate biomarkers. Such markers may refine the prognostic stratification of patients and the diagnostic pathway, as well as providing an objective assessment of changes in disease activity in response to future therapeutic agents. Studies are being extended to the spinal cord, and the application of neuroimaging to unaffected carriers of highly penetrant genetic mutations linked to the development of ALS offers a unique window to the pre-symptomatic landscape.

## Introduction

Amyotrophic lateral sclerosis (ALS; also known as motor neuron disease or Lou Gehrig’s disease) is an adult-onset neurodegenerative disorder of the motor system and its wider connections, characterized pathologically by loss of upper motor neurons of the primary motor cortex and corticospinal tract (CST) projections, together with lower motor neuron loss in the spinal anterior horns and pontomedullary nuclei [1]. Cytoplasmic inclusions of ubiquitinated TDP-43 are the molecular hallmark of 98 % of cases, with a clinical syndrome unified by progressive and irreversible muscle denervation, and death typically arising from neuromuscular respiratory insufficiency. It appears that there are multiple and diverse upstream molecular routes to a final common clinical syndrome [2•].

There is no highly effective disease-modifying treatment for ALS. Despite a median survival from symptom onset of 3–4 years, the syndrome of ALS is surprisingly heterogeneous. Symptomatic onset appears to be focal, typically affecting either the distal limb or bulbar territories, with much rarer respiratory presentation. Subsequent spread of symptoms is not random, but appears to be defined by neuroanatomy with both contiguous and network propagation of pathology [3, 4•]. There is a striking variability in the clinical burden of upper motor neuron (UMN) and lower motor neuron (LMN) signs. Extremes of LMN involvement have been historically termed as progressive muscular atrophy (PMA) and, a very rare UMN-only condition, primary lateral sclerosis (PLS). Both variants are associated with slower rates of progression but are considered part of the spectrum of ALS, which may be encompassed by the broader term, motor neuron disease.

Charcot’s pivotal observation was the additional “*deuteropathic*” involvement of the CST in ALS, marking it out from the normally isolated “*protopathic*” LMN involvement of other peripheral neuromuscular disorders. He did not consider pathology above the brainstem, and it is striking that macroscopically, the primary motor cortex in ALS may appear unremarkable despite severe motor disability. Dedicated post mortem studies of the brain were rare until many decades later when motor pathway involvement was clear [5], even in those without clinical UMN signs [6]. Post mortem neuropathological research has suggested cerebral histopathological ‘stages’ in ALS depending on the extent of TDP-43 pathology [7•].

Although developments in neuropsychology [8, 9], molecular histopathology [10] and genetics [11, 12] have confirmed beyond doubt that ALS lies on a spectrum with frontotemporal dementia (FTD), in fact, the clinical evidence was in the observations of the earliest pioneers of clinical neurology [13]. Overt dementia affects up to 15 % of ALS patients, and is typically an early feature, associated with more rapid physical progression (termed ALS-FTD), though more subtle executive and behavioural dysfunction affects a much larger group of those with ALS, and is also an adverse prognostic sign [14]. ALS is recognised as a multisystem disorder and the link between classically motor and frontal lobe structures can be understood in the context of human evolution [15].

The ability to study the structure and function of the brain in vivo has been a major driver of the rapid advances in clinical neuroscience. ALS superficially appears dominated by its peripheral, more visible clinical manifestations, namely muscle wasting. Electromyography (EMG) has limited sensitivity for detecting sub-clinical denervation caused by loss of LMNs, but remains the current gold standard [16]. The re-formulation of ALS as a fundamentally central nervous system disease pathologically has been underpinned by the application of neuroimaging to the brain, and more recently, also the spinal cord. Findings from neuroimaging have so far typically reflected the insights from post mortem neuropathological study, but also provided new insights through longitudinal studies and network analyses. It is essential to be aware of the limitations of these two techniques, which may nonetheless be regarded as complimentary in furthering our understanding of the pathogenesis in ALS (Table 1).

**Table 1:** Key characteristics of histopathological assessment versus neuroimaging

	Histopathology	Neuroimaging
Setting	Post mortem	In vivo
Scale	Micro: single neurons and synapses	Macro: brain regions and pathways
Scope	Sampled areas	Whole-brain, unbiased
Quantification	By examiner	Semi-automated processing
Timing	Always cross-sectional, typically end-stage disease and with potential for confounding by agonal state	Cross-sectional or longitudinal in symptomatic cases, and in familial cases also pre-symptomatic
Specific potential	Assessment of: - Cell loss (type, layer, quantification) - Protein inclusions - Molecular characterization	Assessment of: - Functional reorganisation at the systems-level - Network properties and efficiency

A coincident emerging research priority of establishing biomarkers in ALS applicable to therapeutic trials has independently brought neuroimaging to the forefront [17•]. ALS remains a fundamentally clinical diagnosis, and one in which delays of 12 months are commonplace. Diagnostic biomarkers, particularly for occult UMN involvement, would have clear value, but more so, biomarkers capable of stratifying patients for therapeutic trials, where heterogeneity may have been an important factor in the failure of drug studies to date. In addition, objective markers of disease activity and progression, rather than the current reliance on rate of change of functional rating scores (e.g., the revised ALS Functional Rating Score, ALSFRS) and survival as outcome measures, would permit faster ‘no-go’ decisions in therapeutic studies [18].

## The Dawn of Functional Neuroimaging: SPECT and PET

**Fig. 1:**
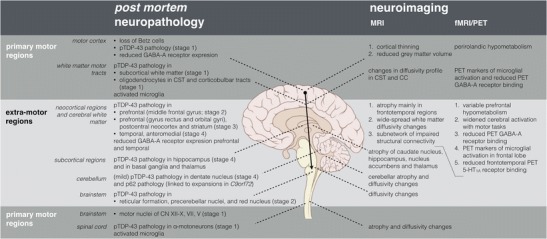
Summary of the pathogenic insights from post mortem histopathological and neuroimaging research in ALS. The findings in the primary motor regions are separately shown from the findings in extra-motor regions. The primary motor regions are defined as the motor cortex (precentral gyrus and paracentral lobule or Brodmann area 4 and 6), the major white matter tracts (corticospinal tracts [CST] and corpus callosum [CC]), the motor nuclei in the brainstem and motoneurons arising from the spinal cord anterior horns. The extra-motor regions are here defined as all other brain regions. TDP-43 pathology is defined according to the post mortem histological stages proposed by Brettschnieder and colleagues [7•]. *pTDP*-*43* phosphorylated, 43-kDa TAR DNA-binding protein, *CST* corticospinal tracts, *CC* corpus callosum, *CN* cranial nerves, *PET* positron emission tomography, *GABA*-*A receptor* gamma-aminobutyric acid receptor type A, *5*-*HT1A receptor* 5-hydroxytryptamine receptor type 1A

In retrospect, the early observations using single-photon emission computed tomography (SPECT) of reduced frontotemporal binding in ALS patients with dementia [19], are entirely predictable with the modern recognition of an ALS-FTD spectrum. The earliest positron emission tomography (PET) studies of cerebral glucose metabolism noted widespread reductions, most obvious not only in those with a high burden of UMN clinical signs [20], but also in relation to more subtle neuropsychological deficits [21, 22]. PET brain activation studies in ALS began to consider the widened ‘boundary shift’ of cerebral activation in relation to motor tasks [23] (Fig. 1). Aside from a clear indication of pathological effects beyond the primary motor cortex, this opened a wider debate that persists today, over the contribution of a disease-related wider recruitment of surviving neurons (perhaps involving plasticity mechanisms) versus a loss of local containment, e.g. through an inhibitory interneuronopathy. Finally, there has been renewed interest in the value of regional patterns of altered fluorodeoxyglucose (FDG) PET as part of the diagnostic pathway in ALS [24, 25].

## Ligand PET Studies

The unrivalled sensitivity of PET has been exploited to understand aspects of the molecular pathology of ALS using radioligands, specifically neuroinflammation, inhibitory cortical influences and serotonin receptor changes (Fig. 1).

### Neuroinflammation

The involvement of microglia and other neuroinflammatory processes in all neurodegenerative disorders is dogged by the issue of primary (pathogenic) versus secondary (reactive, possibly modulatory) involvement. It is clear that the situation is nuanced, possibly with a switch in core microglial function in early versus established disease phases [26, 27]. Until the development of PET ligands specific to activated microglia, evidence for neuroinflammation was largely derived from post mortem histopathology [28, 29]. In vivo studies with the first generation ligand PK11195 revealed evidence for activated microglia in both motor and frontal lobe regions [30]. Furthermore, the signal in both thalamic and primary motor cortex was strongly related to clinical UMN burden. Both findings were confirmed by a more recent study using a ligand with greater specificity [31]. Application of this ligand to rare unilateral forms of ALS (clinically termed Mills’ syndrome) have confirmed topographically localized microglial activation [32], also demonstrated histopathologically [33].

### Cortical Inhibitory Function

Paired stimulation studies using transcranial magnetic stimulation (TMS) have revealed the cortex to be hyperexcitable in ALS [34, 35]. This appears to be quite specific [36], and may predate the onset of symptoms [37]. This supports excitotoxic theories of ALS, which is also compatible with loss of inhibitory GABA-ergic interneuronal influences [38]. PET studies with the GABA-A receptor radioligand flumazenil demonstrated widespread reductions in binding [39], consistent with a loss of inhibitory interneurons, and this was confirmed histopathologically [40, 41]. Moreover, a study comparing sporadic ALS cases with a uniformly more slowly progressive familial form of the condition (matched for clinical features other than disease duration), showed preservation of binding in motor regions of the latter [42], supporting a role for this cell type in pathogenesis.

### Serotonergic Function

PET studies in both ALS [43], and later FTD [44], using a ligand with affinity for the serotonin (5-HT_1A_) receptor, showed marked frontotemporal reductions in binding, confirmed histopathologically in FTD patients [45]. This receptor may be a marker of pyramidal cell loss, or have a more specific role in motor neuronal control or cognition with relevance to both ALS and FTD, and warrants further study.

## Clinical MRI

The principle role of clinical magnetic resonance imaging (MRI) has been in the exclusion of structural pathology mimicking clinical UMN and LMN involvement, principally in the spinal cord [46]. The ubiquity and non-invasive nature of MRI has supported its continued development and a leading role in ALS biomarker discovery [47]. The first application of MRI to a small group of ALS patients highlighted corticospinal tract hyperintensity, particularly visible on T2-weighted sequences [48]. It is neither sensitive nor specific for ALS, though similar changes seen in hepatic failure may provide a clue to common mechanisms of oxidative stress and selective vulnerability [49].

## Advanced Structural MRI

The development of automated segmentation and analysis tools (voxel- and surface-based morphometry), and new acquisition sequences focused on the directionality of the movement of water (diffusion tensor imaging, DTI), have allowed unprecedented in vivo neuropathological study of the brain in ALS (Fig. 1).

### Grey Matter

One of the first automated, quantitative, post-processing imaging tools was voxel-based morphometry (VBM), allowing group comparisons of grey and white matter density profiles to provide an indication of regional brain atrophy patterns. The initial VBM studies in ALS showed diverging results, ranging from focal atrophy in motor regions (precentral gyrus) [50], to widespread atrophy in mostly frontal and temporal regions sparing involvement of the motor cortex [51]. These variable results seem to have been caused by differences in image processing algorithms and statistical models [52].

With increasing patient numbers, however, subgroup analyses comparing bulbar- versus limb-onset patients have shown different atrophy patterns within the motor strip in correspondence with functional disabilities [53•, 54•]. These results are suggestive of focal degenerative cortical changes. Also, the comparison between cognitively impaired and unimpaired patients has demonstrated different patterns of atrophy in which impaired patients show more widespread atrophy extending to extra-motor regions, defined as anatomical regions outside the primary motor regions, mainly in the frontotemporal lobes [55, 56].

Besides VBM, another technique to quantify grey and white matter volumes has emerged, based on the reconstruction of the boundaries between grey and white matter. This technique is referred to as surface-based morphometry (SBM), or cortical thickness measurement and has been successfully applied in ALS. Where VBM is typically based on group comparisons, SBM can provide individual morphometry measures and more detailed information on potential shape alterations. SBM studies have consistently demonstrated cortical thinning in the precentral gyrus or primary motor regions in patients with ALS [57, 58]. Studies comparing volume, thickness and surface area of the cortical mantle have repeatedly shown that cortical thickness has the greatest sensitivity to the disease-related changes in ALS [56, 57]. In addition, similar to what was demonstrated in VBM studies, focal thinning of the motor cortex mirrors clinical features of ALS [58, 59•].

Extra-motor involvement has also been shown more pronounced in patients with additional cognitive deficits and along the course of disease in ALS [60]. This extra-motor cortical thinning is probably the imaging correlate of the widespread degenerative changes, which have also been demonstrated in postmortem studies [61]. It may be important to quantify the extent of UMN and extra-motor involvement since the extent of neuronal involvement correlates with survival, with ALS-FTD typically showing a worse prognosis compared to isolated lower or upper motor neuron phenotypes such as PMA and PLS [62].

In addition to cortical grey matter, recent structural imaging studies have focused specifically on the sub-cortical structures in ALS using VBM- or SBM-based analyses. The caudate nucleus, hippocampus and nucleus accumbens were particularly notable as showing progressive atrophy over time [63, 64], and also the thalamus [54•], in keeping with the earlier observation of intense microglial infiltration of this structure [30]. Such observations are in line with post mortem studies [61, 65], so that neuroimaging now holds the unique promise to reveal the sequence of events along the course of disease, and not just the end stage.

Cerebellar involvement in ALS has gained increased recognition in the past years [66], and structural imaging has revealed unique cerebellar atrophy patterns in ALS, ALS-FTD and FTD, in relation to neuropsychiatric and motor characteristics [67]. This is in alignment with neuropathological findings showing ubiquitin-positive inclusions in the cerebellum, particularly in the granular layer in ALS and FTD [68]. The cerebellum, as a relay station that includes multiple reciprocal cortico-cerebellar connections, appears not to escape disease-related degenerative changes in ALS (akin to the thalamus), despite the lack of overt clinical manifestations.

### White Matter

The most extensively applied technique to study the white matter changes in the ALS brain is diffusion tensor imaging (DTI), which makes use of the typical microstructural organization of white matter as parallel oriented fibres. By assessing the diffusivity characteristics of brain tissue, it becomes possible to identify white matter and assess its integrity. Intact white matter will restrict diffusion of water parallel to the fibre direction, while damaged white matter will cause less restricted diffusivity (quantifiable using measures such as reduced fractional anisotropy, FA or increased mean diffusivity, MD). Based on the main diffusion direction, it is possible to track the course of white matter tracts, which is referred to as tractography. Tractography allows reconstruction of the white matter tracts and thus the brains structural connectivity [69].

It is recognised that the CSTs and corpus callosum (CC) in ALS, as the main white matter motor tracts, have altered diffusivity characteristics compared to healthy controls [17•]. The FA is reduced with an increase in MD, and the more sensitive measure of radial diffusivity (RD) [70, 71]. This altered diffusivity profile might be simply due to loss of white matter fibres as a result of the neurodegenerative process, but may also be compatible with the microglial activation seen in vivo using PET [30, 31] and post mortem histopathology [72]. Currently, there are no systematic studies comparing diffusivity changes in the ALS brain with histopathological white matter changes but this is an active area of research in order to understand better the tissue correlates for DTI changes [73], including in relation to iron deposition [74]. Despite the fact that DTI studies have consistently demonstrated the involvement of the CST at a group level, its diagnostic accuracy for individual ALS patients is currently insufficient [75]. The heterogeneity of CST involvement in cases of ALS without clinical UMN involvement has also been demonstrated in neuropathological studies [76].

In line with the widespread grey matter atrophy, white matter changes have also been shown to extend beyond the primary motor tracts in ALS. Voxel-based DTI studies demonstrated this and, in parallel to patterns of grey matter atrophy, these changes were extensive [77], particularly in patients with cognitive impairment [55, 78]. A large longitudinal study suggested that MRI is sensitive to extensive grey matter changes in ALS [54•].

More recent tract-based analyses, albeit at group rather than individual level, support the view that these widespread white matter changes make up a sub-network of impaired connectivity, typically involving primary and secondary motor connections [79, 80]. Over time, this sub-network of impaired connectivity has been shown to expand, suggesting a network basis for spread of pathology [4•]. Neuropathological studies have shown that phosphorylated TDP-43 can be more or less distributed in the brain, defined as histopathological (rather than clinical) stages [7•]. It does not appear however that every ALS patient will eventually proceed to develop all histopathological stages, and there is particular evidence for the influence of genotype in this respect. For example, it has been shown that ALS patients associated with hexanucleotide repeat expansions in *C9orf72* are more prone to develop ALS with cognitive impairment, which is also related to more extensive cerebral histopathological change [81]. On the contrary, ALS linked to mutations superoxide dismutase (*SOD1*) [82], or the more recently discovered *CHCH10* mutations [83], typically develop a pure motor phenotype in which pathology stays confined to motor regions. Neuroimaging offers unique potential for exploration of in vivo pathological staging of disease [84•], that can be integrated with efforts at clinical staging [85].

## Functional MRI

Blood oxygenation level-dependent (BOLD) MRI has been a valuable tool for the study of brain function. Functional MRI (fMRI) acquisition during motor and cognitive tasks in ALS has now developed into studying the coherence of regional signals under resting-state conditions [86], which is particular suited for a physically disabling condition such as ALS where it is challenging to standardize motor responses during task-based MRI.

### Task-Based fMRI

In line with results from activation PET [23], task-based fMRI studies in ALS have demonstrated increased cortical activation associated with motor processing [87, 88]. Task-based fMRI studies can provide unique insights into processes like functional reorganization, which are most easily explained as recruitment of surviving neurons in an attempt to compensate for structural loss. Besides motor tasks, the functional response to cognitive tasks has also been studied in ALS, including using anti-saccade task of executive dysfunction [89]. A notable recent study reported prefrontal activation abnormalities related to letter fluency in patients with clinically LMN-only form of ALS [90], providing further evidence for a continuum of cerebral involvement across the range of the ALS syndrome, independent of clinical UMN involvement.

### Task-Free (Resting-State) fMRI

Resting-state functional MRI (rs-fMRI) uses the basal brain activity to define functional connectivity between brain regions based on the degree of synchronization of low frequency oscillations [91, 92]. Both regional, as well as whole-brain approaches have been used to study functional connectivity in ALS. Resting-state networks were first investigated in ALS patients using independent component analysis [93]. Of the five resting-state networks extracted, ALS patients showed reduced functional connectivity in the default mode and sensorimotor networks in ALS. The default mode network comprises medial frontal regions, parietal regions, inferior temporal gyrus, cingulate cortex and precuneus, and is consistently deactivated during the performance of cognitive tasks [94, 95]. The sensorimotor network is composed of the primary motor cortex, anterior cingulate cortex, somatosensory regions and auditory cortex [96, 97].

*Reduced* functional connectivity in the motor network was replicated in other studies [98, 99]. However, subsequent studies have found *increased* functional activity within and beyond the motor and premotor cortex, despite reduced structural connectedness [100], or a combination of increased and decreased functional coherence within cortical sub-regions [101–103]. A recent study aiming to address the link between functional and structural connectivity in ALS assessed all affected structural connections in ALS and showed an overall positive correlation between structural and functional connectivity [104]. A paradoxical relation between structural and functional connectivity exists in specific sub-regional connections which may be related to compensatory mechanisms, but perhaps also drive pathogenesis more directly [100].

In line with previous PET but also structural imaging studies, rs-fMRI has shown connectivity changes in the default mode and frontoparietal networks in relation to cognition and behaviour in ALS. Enhanced parietal connectivity was associated with the clinical and cognitive deficits of the patients and suggested to be a compensatory mechanism [103]. In line with this study, a study comparing both FTD (behavioural variant) and ALS patient groups showed increased signal in the posterior cingulate cortex in FTD patients that were suppressed in ALS patients. Overall, however, functional connectivity patterns in ALS and FTD were found to be strikingly similar as may be expected in diseases belonging to the same clinicopathological spectrum [105]. It is hoped that techniques like magnetoencephalography (MEG), with a much higher temporal resolution compared to fMRI, will help to further unravel functional connectivity changes in ALS [106].

## Magnetic Resonance Spectroscopy

The metabolite content of tissue can be explored using magnetic resonance spectroscopy (MRS). The most robustly distinguishable metabolites in the brain with proton MRS are N-acetyl aspartate (NAA, a marker of neuronal density), choline (Cho, a marker of membrane integrity) and creatine (Cr, a marker of cellular energetics). NAA is typically expressed as a ratio with one or both of Cho and Cr. In ALS, NAA reductions in the primary motor cortex have been among the most consistent findings, initially using single-voxel approaches [107], and more recently, whole-brain analysis [108]. Glutamate and glutamine increases in the brainstem have supported excitotoxic theories of ALS [109], with equally consistent reductions in GABA reported more recently [110].

## Advanced Spinal Cord MRI

Supported by studies using *SOD1* transgenic mouse models of ALS, which are necessarily biased towards the study of LMNs, the concept of ALS as a ‘dying back’ pathological process from the LMNs to involve the CSTs and wider brain has persisted. This is despite recognition of the overlap between ALS and FTD, and with cognitive involvement preceding that of the downstream motor system in a significant proportion of cases. The study of spinal cord pathology in vivo would have particular value in furthering knowledge about pathological spread and the variation in UMN and LMN involvement clinically. Issues with resolution, physiological movement and the close proximity of bone have made the application of MRI challenging [111]. Nonetheless, it has been possible to demonstrate structural changes in the cord in ALS, as well as show sub-clinical involvement of sensory pathways [112•].

## Genetic Imaging

A diverse group of genetic mutations has been linked to a small proportion of cases with the syndrome of ALS [113]. Pathological mutations in *SOD1* and hexanucleotide expansions in *C9orf72* account for over half of the 5 % of ALS cases reporting a clear family history of either ALS or FTD. Whereas cognitive or behavioural involvement is exceptional in *SOD1*-mediated ALS, it is common in *C9orf72* expansion-associated ALS where carriers within the same family may develop either ALS-FTD or pure FTD [114]. MRI studies in ALS patient carriers of *C9orf72* expansions revealed extensive changes, including the involvement of sub-cortical structures such as the thalami and clinically silent regions, e.g. cerebellum, despite attempts to control for the greater cognitive involvement in such patients compared to the sporadic ALS comparator group [115]. Similar more widespread changes in FDG uptake were noted in a PET study of *C9orf72*-related ALS patients [116]. DTI studies in patients homozygous for the ‘D90A’ *SOD1* mutation associated with a consistently slower form of the disease, showed strikingly preserved CST integrity despite similar UMN burden clinically [70], which may reflect altered regional white matter vulnerability.

### Pre-symptomatic Studies

Carriers of genetic mutations linked to the development of ALS offer a unique window to study the pre-symptomatic pathological landscape, both to understand the very earliest changes and to open the possibility of neuroprotection in the longer term [117]. A flumazenil PET study of two pre-symptomatic *SOD1* mutation carriers hinted at left operculum region changes similar to those seen in affected patients [42]. DTI studies in other *SOD1* patients have been conflicting, with one showing bilaterally reduced FA in the region in the internal capsule [118] but another no significant difference [119]. The metabolic profile of the cervical cord in a pre-symptomatic *SOD1* patient study using MRS was more aligned to affected patients than healthy controls [120]. Combined DTI and rs-fMRI studies in pre-symptomatic carriers of FTD-only related genes (*MAPT* and *PRGN*) found evidence of both structural and functional change [121], with the expectation that there will be similar findings in *C9orf72* expansion carriers when the results of such studies are known.

It has also been possible to explore the pre-symptomatic period by applying MRI to the transgenic *SOD1* mouse model of ALS. This has revealed T2-weighted intensity changes in brainstem nuclei, corresponding to vacuolation and microglial infiltration histologically [122•], and which could be modified with anti-inflammatory therapy [123].

## Multimodal Analyses

At present, the variation in brain structure and function has made the application of advanced MRI techniques limited to group-level differences. However, it is hoped that a combination of measures from different acquisitions can provide a signature applicable at the individual patient level. Support for this discriminatory improvement has come from combined studies of structural and functional MRI [100], and from combined DTI and MRS [124, 125]. It has also been possible to link levels of neurofilament light chain in the CSF of ALS patients to the structural integrity of the CSTs as measured by DTI [126], linking biochemical and neuroimaging biomarkers directly.

## Standardization and Harmonization

In 2010, the Neuroimaging Society in ALS (www.NiSALS.org) was formed by a group of international scientists with a focus on the application of neuroimaging to the study of ALS. It has met annually to consider new developments in the field, but also to develop a standardized protocol for use as a source of outcome measures in therapeutic trials [127]. The establishment of a data repository has allowed the first multi-centre studies, which have confirmed the feasibility of MRI in this setting despite the undoubted major challenges of intra- and inter-site variability.

## Concluding Remarks

Neuroimaging has brought unparalleled in vivo pathological insights into ALS, now also starting to include the pre-symptomatic landscape. The precedent set in Alzheimer’s disease by the development of an in vivo radioligand for the intracellular molecule tau [128], opens the possibility of a TDP-43 equivalent that might have particular value in the pre-symptomatic period. In addition, core mechanisms may be revealed that have implications for future therapeutics in ALS and neuroimaging has shown its great potential for generating biomarkers that can be used to efficiently test them. Combining traditional neuropathology with the power of in vivo neuroimaging data offers the best opportunity to further understanding of pathogenesis in ALS.
